# Rapamycin Antagonizes BCRP-Mediated Drug Resistance Through the PI3K/Akt/mTOR Signaling Pathway in mPRα-Positive Breast Cancer

**DOI:** 10.3389/fonc.2021.608570

**Published:** 2021-04-12

**Authors:** Jing Zhang, Jing Hu, Weiwei Li, Chunyan Zhang, Peng Su, Yan Wang, Wei Sun, Xiao Wang, Li Li, Xiaojuan Wu

**Affiliations:** ^1^ Department of Pathology, School of Basic Medical Sciences, Shandong University, Jinan, China; ^2^ Department of Pathology, Qilu Hospital, Shandong University, Jinan, China

**Keywords:** breast cancer, drug resistance, BCa resistance protein (BCRP), membrane progesterone receptor alpha, PI3K/Akt/mTOR, rapamycin, targeted therapy

## Abstract

**Purpose:**

Overexpression of breast cancer (BCa) resistance protein (BCRP) is detected in approximately 30% of BCa cases. BCRP indicates a poor response to chemotherapy, and it has become a classic target to overcome drug-resistant tumor cells. In this study, we aimed to explore the mechanism of BCRP overexpression and a strategy to reverse this overexpression in invasive BCa.

**Methods:**

BCRP expression in BCa tissues was determined by immunohistochemistry. GSE25066 was downloaded from the NCBI GEO database. Western blot was used to determine the expression of key molecules *in vitro*. Cell counting kit-8 assays were used to assess the drug response of BCa cells.

**Results:**

Our results suggested that BCRP is an independent risk factor for BCa. We further established that upon 17α-PG binding, membrane progesterone receptor α (mPRα) promoted BCRP expression *via* the PI3K/Akt/mTOR signaling pathway. mPRα physically interacted with p-Akt1 S473. Moreover, rapamycin, an inhibitor of mTOR complex 1 (mTORC1), downregulated BCRP expression and enhanced the effects of particular drugs, including doxorubicin and paclitaxel.

**Conclusion:**

BCRP is a potential biomarker of poor prognosis in BCa. BCRP expression is regulated by 17α-PG in mPRα-positive BCa cells through the PI3K/Akt/mTOR signaling pathway. Rapamycin might enhance the therapeutic effect of chemotherapy agents in mPRα-positive MDA-MB-453/BCRP cells and might be a therapeutic option for mPRα-positive invasive BCa with BCRP overexpression.

## Introduction

Chemotherapy is considered to be a vital treatment strategy in invasive breast cancer (BCa). However, *de novo* or acquired drug resistance remains the major obstacle of treatment. The human BCa resistance protein (BCRP) localizes to the plasma membrane and pumps a variety of endogenous and exogenous compounds out of the cell. Due to its function, BCRP plays a critical role in BCa drug resistance by increasing the cellular excretion of chemotherapeutics (1–3).

Female sex hormones play key roles in the pathogenesis of BCa. We have reported that progesterone receptor (PR) and estrogen receptor α (ERα) modulate the expression of BCRP, and overexpression of BCRP promotes BCa metastasis (4). This phenomenon partially depends on the classic genomic pathway of steroid nuclear receptor signaling. In addition, all types of steroid hormones have been reported to have rapid, nongenomic effects that occur at the cell membrane or mitochondria (5). Studies have shown that steroid hormones induce rapid effects though several signaling pathways, including the EGFR and MAPK pathways (5–7). Steroid hormone receptors interact with multiple signaling molecules modeling their activation state.

We focused on the nongenomic signaling of PRs. There are three isoforms of membrane progesterone receptors (mPRs) in humans, mPRα, mPRβ, and mPRγ; mPRα is the predominant subtype in invasive BCa and in BCa cell lines (8). We previously reported a positive correlation between mPRα protein expression and lymph node metastasis, suggesting that mPRα is a potential mediator of invasive BCa progression. We also revealed that mPRα promotes breast cancer invasion though matrix metalloprotein (MMP9) by activating the PI3K/Akt signaling pathway (9).

However, the role of mPRα in BCa drug resistance is still elusive. Moreover, its effect on BCRP expression through the rapid, nongenomic signaling pathway has not been reported. This investigation focused on the mechanism by which 17α-PG regulates BCRP expression through the nongenomic pathway.

## Materials and Methods

### Patients and Tissue Samples

In the present research, we obtained a total of 215 invasive BCa samples from the Department of Pathology, Qilu Hospital of Shandong University. Among them, 103 cases from 2007 to 2008 were followed up. Overall survival (OS) referred to the interval from initial treatment until recurrence, metastasis, or death. Other 112 BCa samples were from 2012 to 2014. There was no significant difference in clinicopathological parameters between the two cohorts (**Supplementary Table 1**). This study was approved by the Shandong University Medical Research Ethics Committee (approval number 201401016), and written informed consent was obtained from each patient in accordance with the Declaration of Helsinki. Survival analysis of BCRP were proceed with 103 BCa samples from 2007 to 2008. Correlation analysis of mPRα, p-Akt1 S473, and BCRP were proceed with a total of 150 BCa samples containing 38 samples from 2007 to 2008 and 112 samples from 2012 to 2014.

### Immunohistochemistry

IHC was performed as previously described (10). The slides were incubated with primary antibodies against mPRα (ab75508, 1:500, Abcam), phosphorylated Akt1 (p-Akt1 S473; #4060, 1:500, CST), or BCRP (ab3380, 1:40, Abcam) for 2 h at RT. A semiquantitative scale combining staining intensity and the percentage of positive cells was used to grade protein expression; staining was scored from 0 to 3 (0 = no expression; 1 = weak; 2 = moderate; and 3 = strong), as was the percentage of positive cells (0 < 10%; 1 = 10%–40%; 2 = 40%–70%; and 3 ≥ 70%). Normal breast tissues were used as a positive control. Slides were evaluated and scored by two pathologists separately.

### Cell Culture and Reagents

The human (BCa) cell lines MDA-MB-453 and MDA-MB-231 were obtained from American Type Culture Collection (Rockville, MD, USA) and cultured following the manufacturer’s recommendations. MDA-MB-453 and MDA-MB-231 cells are negative for nuclear progesterone receptor (nPR). The cell counting kit-8 (CCK8) was purchased from BestBio Biotechnology (Shanghai, China).

The plasmid pEGFP/C-BCRP harboring full-length BCRP cDNA was constructed and stored in our laboratory; this plasmid was transfected into MDA-MB-453 and MDA-MB-231 cells to establish the MDA-MB-453/BCRP and MDA-MB-231/BCRP stable cell lines as previously reported (11). The siAkt was purchased from GenePharma (Shanghai, China) and Akt inhibitor of perifosine was obtained from MedChemExpress (Shanghai, China).

### Western Blotting

Western blot was performed as previously described.10 The membranes were incubated overnight with antibodies against PI3K (#YT3713, 1:500, ImmunoWay), phosphorylated PI3K (p-PI3K Y467/199; #YP0224, 1:500, ImmunoWay), mTOR (#YT2915, 1:500, ImmunoWay), phosphorylated mTOR (p-mTOR S2448; #YP0176, 1:500, ImmunoWay), Akt1 (#2920, 1:1,000, CST), phosphorylated Akt1 (p-Akt1 S473; #4060, 1:1,000, CST), phosphorylated 4E-BP1 (p-4E-BP1 Ser65; #9451, 1:1,000, CST), mPRα (ab75508, 1:1,000, Abcam), BCRP (ab3380, 1:500, Abcam), and β-actin (TB346894, 1:1,000, ZSGB-Bio). Immunoreactivity was visualized using an enhanced chemiluminescence kit (Millipore, Darmstadt, Germany). ImageJ was used for quantitative analysis.

### Coimmunoprecipitation

Cells in 10 cm^2^ plates were lysed in ice-cold RIPA lysis buffer. Lysates containing 500 μg of total protein were incubated with specific antibodies (2 μg) for 18 h at 4°C with constant rotation. Then, 20 μl of protein A+G agarose beads was added to bind the protein-antibody complexes, and the mixtures were incubated at 4°C overnight, followed by centrifugation at 2,500 rpm for 5 min at 4°C. The supernatants were discarded, and the precipitates were washed five times with PBS. After the final wash, the precipitates were pelleted by centrifugation and boiled in SDS sample buffer for western blot analysis. IgG was used as a negative control.

### Statistical Analysis

Data analysis was performed with SPSS version 18.0. The correlations between BCRP expression and the clinicopathological parameters as well as among mPRα, p-Akt1 and BCRP expressions were evaluated by the Pearson chi-square tests.

The correlations between BCRP expression and overall survival curves were plotted by the Kaplan–Meier method and compared with the log-rank test. Univariate and multivariate Cox regression analyses were used to evaluate survival data. Statistical significance was considered when P values were <0.05.

## Results

### Determination of the Prognostic Significance of BCRP in Invasive BCa

**Figure 1:**
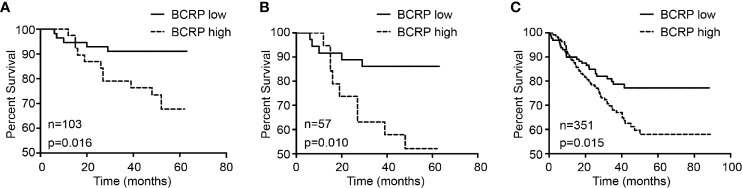
BCRP was related to poor prognosis of BCa. **(A)** The correlation between BCRP expression in BCa tissue and overall survival (n = 103, p = 0.016, Kaplan-Meier survival analysis, log-rank test). **(B)** The correlation between BCRP expression in BCa tissue and overall survival among patients with lymph node metastasis (n = 57, p = 0.010, Kaplan-Meier survival analysis, log-rank test). **(C)** The correlation between BCRP expression in BCa tissue and overall survival among patients with lymph node metastasis from GSE25066 (n = 351, p = 0.015, Kaplan-Meier survival analysis, log-rank test).

BCRP expression in primary BCa was evaluated by IHC. Patient characteristics are shown in **Table 1**. Univariate and multivariate analyses with Cox proportional hazards regression models were used to explore the associations between BCRP expression and OS. As shown in **Table 2**, the univariate analysis suggested that BCRP expression, tumor grade and lymph node metastasis (LNM) were significantly correlated with the OS of BCa patients (**Table 2**, P<0.05). Multivariate analysis showed that BCRP expression was an independent prognostic predictor for OS (HR=4.102, 95% CI: 1.395–12.061). Kaplan-Meier plots of OS relative to BCRP expression are shown in **Figure 1A**. We further explored the significance of BCRP expression in LNM-positive BCa patients. Kaplan-Meier analysis showed that BCRP expression was negatively related to OS in LNM-positive patients (**Figure 1B**). Similar results were obtained with the data in GSE25066 (**Figure 1C**).

**Table 1:** Clinicopathological characteristics of 103 patients with follow up in the present study.

Variable	Count	Percentage (%)
**Age**		
≤ 65	90	87.4
> 65	13	12.6
**Grade**		
I–II	68	66.0
III	35	34.0
**LNM**		
Negative	46	44.7
Positive	57	55.3
**ER**		
Negative	36	35.0
Positive	67	65.0
**PR**		
Negative	32	31.1
Positive	71	68.9
HER2		
Negative	84	81.6
Positive	19	18.4
BCRP		
Negative	62	60.2
Positive	41	39.8

**Table 2:** Univariate and multivariate analysis for overall survival (Cox proportional hazards regression model).

Variable	Univariate analysis			Multivariate analysis		
	P	HR	CI (95%)	P	HR	CI (95%)
Age	0.407	2.356	0.3–17.8911			
Grade	0.015	3.512	1.275–9.668	0.29	1.858	0.59–5.847
LN	0.02	11.018	1.448–83.808	0.043	9.412	1.073–82.532
BCRP	0.024	3.395	1.1799.779	0.010	4.102	1.395–12.061
ER	0.061	0.387	0.144–1.043			
PR	0.548	0.732	0.265–2.024			
HER2	0.412	1.607	0.518–4.983			

### Regulation of BCRP Expression by 17α-PG

**Figure 2:**
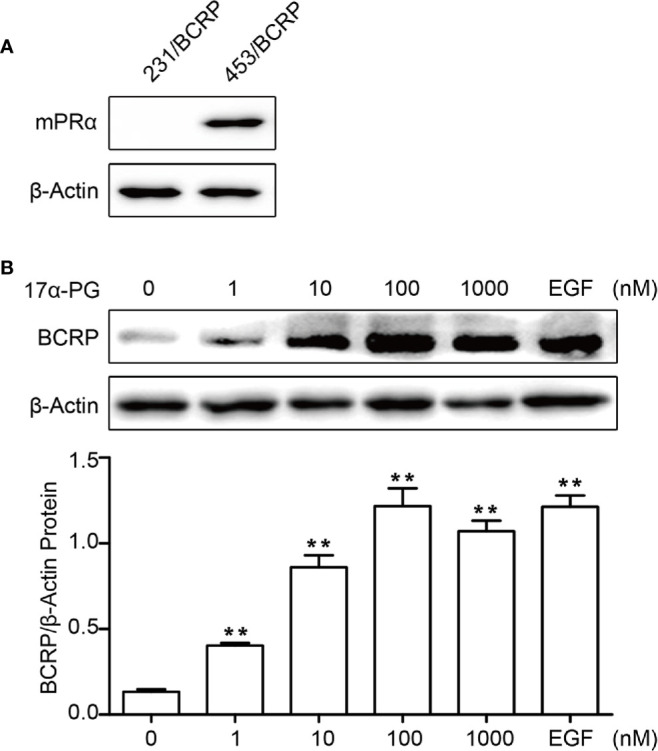
BCRP was regulated by 17α-PG in mPRα-positive BCa cells. **(A)** Western blot analysis of mPRα expression in MDA-MB-231/BCRP and MDA-MB-453/BCRP cells. **(B)** BCRP protein expression in response to treatment with different concentrations of 17α-PG and EGF for 24 h. The data shown in the histogram are the mean ± SD of three independent experiments. *P < 0.05, **P < 0.01, ***P < 0.001.

To elucidate the mechanism regulating BCRP expression in BCa, we treated BCa cells with 17α-PG, a selective agonist of mPRα. As shown in **Figure 2**, the expression of BCRP in mPRα-positive MDA-MB-453/BCRP cells (**Figure 2A**) was upregulated by 17α-PG in a dose-dependent manner, with EGF as a control (**Figure 2B**). There was no detectable change in mPRα-negative MDA-MB-231/BCRP cells (data not shown). These data suggest that the induction of BCRP expression by 17α-PG is dependent on mPRα status.

### Interaction Between mPRα and p-Akt1 S473 in MDA-MB-453/BCRP and MDA-MB-231/BCRP Cells

**Figure 3:**
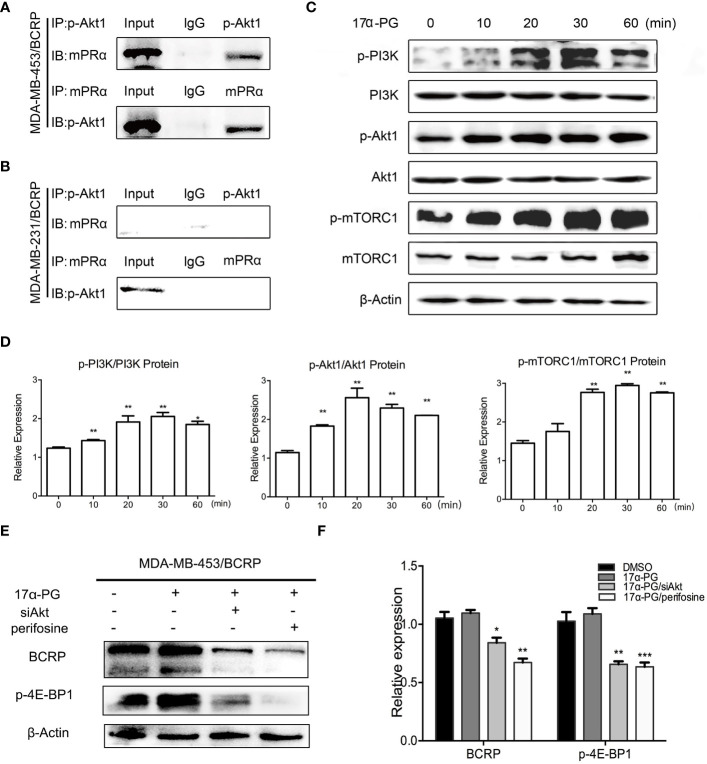
17α-PG activated PI3K/Akt/mTOR signaling through the interaction between mPRα and p-Akt1 S473. **(A, B)** Interaction between mPRα and p-Akt1 S473 in MDA-MB-453/BCRP **(A)** and MDA-MB-231/BCRP **(B)** cells. Samples (500 μg) were subjected to CoIP using an anti-mPRα antibody (5 μg) and anti-p-Akt1 antibody (5 μg). Immunoprecipitated lysates were then analyzed by western blotting using anti-p-Akt1 and anti-mPRα antibodies. **(C, D)** PI3K/Akt/mTOR signaling in response to 17α-PG in MDA-MB-453/BCRP cells. The cells were starved for 48 h and then treated with 1,000 nM 17α-PG for 0, 10, 20, 30, and 60 min. Western blotting was performed to check the activation of PI3K, Akt1, and mTOR. **(D)** The data shown in the histogram are the mean ± SD of three independent experiments. *P < 0.05, **P < 0.01, ***P < 0.001. **(E, F)** The rescue test of PI3K/Akt/mTOR signaling in response to 17α-PG in MDA-MB-453/BCRP cells. The cells were starved for 48 h and then treated with 1,000 nM 17α-PG combined with siAkt or perifosine. Western blotting was performed to check the activation of p-4E-BP1 and expression of BCRP. **(F)** The data shown in the histogram are the mean ± SD of three independent experiments. *P < 0.05, **P < 0.01, ***P < 0.001.

We previously showed that mPRα and p-Akt1 S473 are positively correlated in invasive BCa. This correlation leads to the hypothesis that 17α-PG might regulate BCRP expression though PI3K/Akt/mTOR signaling. To explore the interactions between mPRα and p-Akt1 S473, we used antibodies against mPRα and p-Akt1 S473 to immunoprecipitate proteins from total cell lysates of MDA-MB-453/BCRP and MDA-MB-231/BCRP cells and subsequently confirmed the identity of the immunoprecipitated proteins by western blot. The results showed an interaction between mPRα and p-Akt1 S473 in MDA-MB-453/BCRP cells (**Figure 3A**) but not in MDA-MB-231/BCRP cells (**Figure 3B**).

### Activation of PI3K/Akt/mTOR Signaling by 17α-PG in MDA-MB-453/BCRP Cells

To further prove whether the PI3K/Akt/mTOR signaling pathway is involved in the 17α-PG action in mPRα-positive cells, the cells were treated with 1,000 μM 17α-PG for 0, 10, 20, 30, and 60 min. Expression levels of PI3K, p-PI3K Y467/199, Akt1, p-Akt1 S473, mTOR, and p-mTOR S2448 were detected in fresh protein samples by western blot analysis. As revealed in **Figures 3C, D**, the levels of phosphorylated signaling molecules were markedly upregulated compared with the total levels of each protein at 20 min after 17α-PG treatment. While after treatment the cells with Akt specific inhibitor perifosine or siRNA targeted at Akt, the expressions of p-4E-BP1 and BCRP were both decreased (**Figures 3E, F**). These data suggest that 17α-PG might induce rapid effects, such as the regulation of BCRP expression, through activation of the PI3K/Akt/mTOR pathway in mPRα-positive MDA-MB-453/BCRP cells.

### Positive Correlations Among mPRα, p-Akt1 S473, and BCRP in BCa Tissue

**Figure 4:**
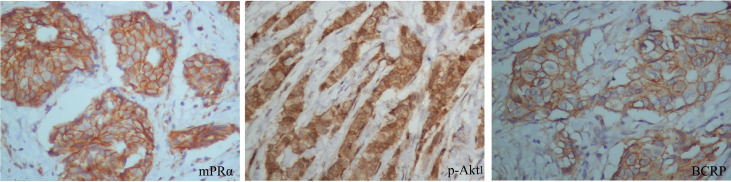
BCRP expression was positively related to mPRα and p-Akt1 S473 in breast tissue. Representative BCa specimens positive for mPRα, p-Akt1 S473, and BCRP, as detected by IHC. Photographs were taken at ×400 magnification. Invasive BCa positive for mPRα (left), p-Akt1 S473 (middle), and BCRP (right). Note that in BCa, mPRα and BCRP were detected at the membrane and/or in the cytoplasm, while p-Akt1 S473 was mainly detected in the cytoplasm.

We next examined whether p-Akt1 S473 and the mPRα-mediated regulation of BCRP are physiologically relevant in human breast tissues. We verified the correlations among mPRα, p-Akt1 S473 and BCRP in primary breast invasive cancer by IHC. Notably, BCRP overexpression was significantly associated with mPRα (P=0.002) and p-Akt1 S473 (P=0.048) by IHC in our cohort. Representative images are shown in **Figure 4**. The positive correlation is shown in **Table 3**.

**Table 3:** Expression of mPRα, p-Akt1, and BCRP in invasive breast cancer.

		*n*	BCRP		*P-value*
			positive	negative	
mPRα	positive	63	28	35	0.009
	negative	87	21	66	
p-Akt1	positive	67	29	38	0.048
	negative	83	22	61	

### Effect of Rapamycin on BCRP Expression

**Figure 5:**
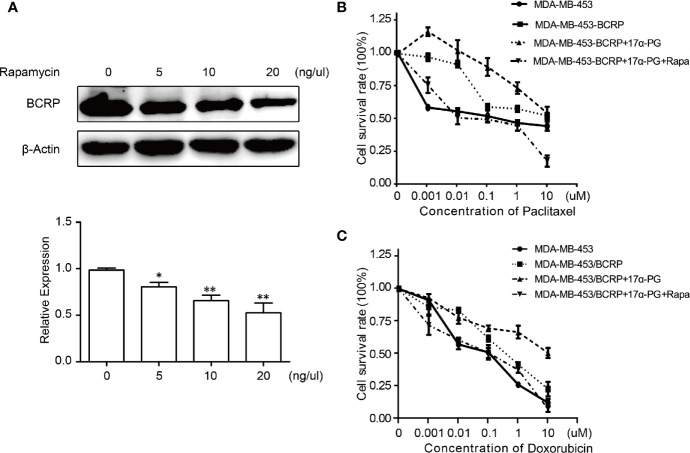
Rapamycin reversed 17α-PG-mediated BCRP expression and drug resistance. **(A)** The concentration-dependent inhibition of BCRP expression by rapamycin. The cells were incubated with 0, 5, 10, or 20 ng/μl rapamycin for 24 h. Relative expression data are shown in the histogram with β-actin as the loading control. The results are from three independent experiments. *P < 0.05, **P < 0.01, ***P < 0.001. **(B, C)** The survival rate of MDA/MB-453/BCRP cells exposed to doxorubicin **(B)** and paclitaxel **(C)** combined with rapamycin, as determined by CCK-8 assays. Cells were seeded in 96-well plates at 5 × 10^3^ cells per well, and both cell lines were preincubated with rapamycin for 24 h. Then, the cells were treated with a concentration gradient of doxorubicin and paclitaxel for another 48 h. The points indicate the percentage relative to control MDA-MB-453 cells. Data are the mean of triplicate determinations, and the SD is indicated by the bars. *P < 0.05, **P < 0.01, ***P < 0.001.

To further confirm the above hypothesis, BCRP expression was assessed in MDA-MB-453/BCRP cells following treatment with rapamycin, a selective antagonist of mTOR signaling. As indicated in **Figure 5A**, BCRP overexpression was suppressed by rapamycin in dose-dependent manner in mPRα-positive MDA-MB-453/BCRP cells. This finding suggests that BCRP expression depends on activation of the PI3K/Akt/mTOR signaling pathway.

### Effect of Rapamycin on Sensitivity of MDA/MB-453/BCRP Cells to Doxorubicin and Paclitaxel

To clarify the importance of the PI3K/Akt/mTOR signaling pathway in BCRP-mediated drug resistance in mPRα-positive BCa, we examined the survival rate of rapamycin-treated MDA-MB-453/BCRP cells. Compared with MDA-MB-453/BCRP cells treated with 17α-PG alone, the relative survival of MDA-MB-453/BCRP cells treated with 17α-PG combined with rapamycin on doxorubicin or paclitaxel decreased ~15.5-fold and ~58.9-fold, respectively (**Figures 5B, C**). All of these findings confirmed that 17α-PG promotes BCRP expression through the PI3K/Akt/mTOR signaling pathway and that rapamycin significantly enhances the chemosensitivity to doxorubicin and paclitaxel by blocking this pathway in mPRα-positive MDA-MB-453/BCRP cells.

## Discussion

Chemoresistance is a critical challenge of BCa treatment. However, the underlying molecular mechanisms are not yet clear. An increasing number of studies have focused on the reversal of multidrug resistance. Our previous studies systemically demonstrated the mechanisms of the key mediators MDR1 and BCRP of BCa chemoresistance and proposed a reversal strategy (12, 13). In the current work, we found that rapamycin could reverse the chemoresistance mediated by BCRP. Mechanistically, we revealed that 17α-PG activated BCRP expression though PI3K/Akt/mTOR signaling by a physical interaction between mPRα and p-Akt S473.

BCRP belongs to the ATP-binding cassette (ABC) family. The ABC transporters remove substrates from cells against a concentration gradient. Three transporters have been implicated in drug resistance, including the P-glycoprotein efflux pump (P-gp) encoded by the ABCB1 gene, the multidrug resistance-associated protein-1 (MRP-1) encoded by the ABCC1 gene, and BCRP encoded by the ABCG2 gene (14). BCRP is a transmembrane protein located on the apical membrane of epithelial cells such as those of the intestine, liver, kidney, and syncytiotrophoblast, and it transports xenobiotic and endogenous substrates out of cells (15). Normally, BCRP plays a critical role in protecting the organism against exposure to xenobiotics and endogenous chemicals. However, BCRP expression in certain cancers, such as BCa, lung cancer, gastric cancer and leukemia, is likely a reflection of a drug-resistant phenotype (16–18). Considerable efforts have been devoted to determining the role of BCRP in drug resistance in BCa (18). However, the effect of BCRP on survival remains controversial. We believe that the discrepant outcomes might be due to the shorter follow-up time or the small number of cases included in this study. Furthermore, the status of nuclear estrogen receptor (nER), nPR, C-erbB-2, Ki-67, and LNM also plays a vital role in determining the prognosis of invasive BCa.

Apart from the genomic pathway, nongenomic steroid signaling, including by PG, is usually regulated through membrane- or cytoplasmic-localized classic steroid receptors, such as mPRs (19). There has been new insight into the roles of PG based on the identification of mPRs in triple-negative BCa, which lacks nER, nPR, and C-erbB-2 and is insensitive to antihormone and/or targeted anti-C-erbB-2 therapies (20). Progestin can rapidly initiate the PI3K/Akt, Src/Ras/MAPK or JAK2/Stat3 signaling pathway in BCa and mammary epithelial cells, suggesting that mPR is a potential mediator of nongenomic cellular signaling pathways (21–23). mPRs belong to the progestin and adipoQ receptor (PAQR) family, which contains three isoforms in human, mPRα, mPRβ, and mPRγ (24, 25). Knowledge of the mechanism of mPRα in BCa development and progression has gradually accumulated (26, 27), but its precise role in drug resistance is still unclear.

To explore the mechanisms underlying chemoresistance induced by mPR expression in BCa, we selected mPRα and BCRP for further observation. In our previous research, we found that PG negatively regulated BCRP expression by genomic signaling through nPR. 17α-PG is a selective mPRα agonist and affirms the existence of functional mPRα in cell lines. Further research confirmed that 17α-PG treatment could upregulate BCRP expression through rapid activation of the PI3K/Akt/mTOR signaling pathway in a dose-dependent manner in MDA-MB-453/BCRP cells. Although the relationship between mPRα and the PI3K/Akt signaling pathway has been confirmed (28), no previous reports have shown the interaction between mPRα and BCRP in this pathway. Akt1 is the central member of the PI3K/Akt/mTOR signaling pathway (29). Furthermore, the interaction between mPRα and p-Akt1 S473 was confirmed, suggesting that mPRα regulates BCRP expression though PI3K/Akt/mTOR signaling. The significant association between mPRα and BCRP in BCa tissues further confirmed this regulation.

Neoadjuvant therapy aims to shrink the primary neoplasm for subsequent surgical removal. nER, nPR, and C-erbB-2 are commonly identified as indications for neoadjuvant therapy. PI3K/Akt/mTOR signaling pathway activation is involved in resistance to endocrine therapy and cytotoxic therapy in hormone receptor positive BCa (30). However, the extranuclear hormone receptor status remains to be elucidated. In this research, we found that in comparison with single agent 17α-PG treatment, the combination of an mTOR complex 1 (mTORC1) inhibitor with PG could sensitize mPRα-positive BCa to doxorubicin and paclitaxel.

Rapamycin, a macrocyclic lactone isolated from *Streptomyces hygroscopicus*, was the first rapalog investigated in the clinic (31); it binds to FKBP12, inhibits the mTORC1 complex and blocks mTOR signal transduction in cell growth and proliferation (32). In recent years, the roles of mTOR inhibitors have been extensively studied for their safety and effectiveness. However, the disadvantage of mTOR inhibitors is that they are unlikely to be useful as single agents in most malignancies. Combining this kind of agent with conventional cytotoxic medicines or other targeted therapies has been suggested as a potentially effective way to improve cure rates. Combined treatment with PI3K/Akt/mTOR pathway inhibitors and neoadjuvant agents was found to potentially increase chemosensitivity in ER-positive BCa (33). In this research, we focused on whether the combination of rapamycin with chemotherapy improves the sensitivity of mPRα-positive BCa to chemotherapy. We observed that the upregulation of BCRP was inhibited by rapamycin in a dose-dependent manner. Treatment with rapamycin increased chemosensitivity and reversed BCRP-induced drug resistance in mPRα-positive BCa cells.

These data are in line with previous reports by other groups, including one report that imatinib inhibits BCR–ABL in resistant K562 leukemic cells through posttranscriptional regulation of BCRP expression *via* the PI3K/Akt signaling pathway (34).

From above, studies have shown that BCRP overexpression is one of the markers of poor prognosis in breast cancer. Its expression in subtype of luminal breast cancer was higher than that of triple negative breast cancer (TNBC). While there was no significant difference between HER2 positive breast cancer and TNBC. Luminal type patients can choose endocrine therapy or chemotherapy. While HER2 over-expression is the main drive in breast cancer and anti-HER2 drugs are the main type of therapy for these patients. But TNBC has limited treatment options. At present, traditional chemotherapy is still the main treatment and the curative effect is not very well. In this study, we elaborated the mechanism of rapamycin on sensitizing doxorubicin and paclitaxel in cells of TNBC with mPRα positive, which might provide experimental basis for chemotherapy. But we did not observe the effect in HER2 overexpression breast cancer cells with mPRα positive. This is the limitation of this experiment and we will increase the subtypes of breast cancer cell lines in future research to further observe the mechanism of rapamycin on anti-HER2 drugs.

## Conclusion

BCRP is a potential biomarker for poor prognosis in primary invasive BCa that might be modulated by mPRα through nongenomic regulation *via* the PI3K/Akt/mTOR signaling pathway. Combining an mTORC1 inhibitor with chemotherapy agents sensitizes cells to conventional chemicals and might improve their effectiveness, which may shed light on the use of an mTORC1 inhibitor to overcome resistance in mPRα-positive BCa.

## Data Availability Statement

The original contributions presented in the study are included in the article/**Supplementary Material**. Further inquiries can be directed to the corresponding author.

## Author Contributions

XjW conceived of the idea and wrote the manuscript. JZ and JH cultured the cells. WL, CZ and WS carried out western blot and CCK-8 assays. PS, YW, and XW conducted the IHC. JZ and PS analyzed the data. XjW and LL edited the manuscript. All authors contributed to the article and approved the submitted version.

## Funding

This research was supported by the National Natural Science Foundation of China (NSFC, grant No. 81402181) and the National Natural Science Foundation of Shandong Province (grant No. ZR2019MH069).

## Conflict of Interest

The authors declare that the research was conducted in the absence of any commercial or financial relationships that could be construed as a potential conflict of interest.
