# Supplementary material for: Rapamycin Antagonizes BCRP-Mediated Drug Resistance Through the PI3K/Akt/mTOR Signaling Pathway in mPRα-Positive Breast Cancer
Source: Front Oncol. 2021 Apr 12;11:608570. doi: 10.3389/fonc.2021.608570 (PMC8071953; doi:10.3389/fonc.2021.608570)
Supplement: Supplementary file 1 [file Table_1.docx]

**Supplement Table1 Analysis of clinicopathological parameters of two cohorts**
